# Closed genome sequences of two *Listeria monocytogenes* ST121 strains

**DOI:** 10.1128/MRA.00750-23

**Published:** 2023-09-28

**Authors:** Bienvenido W. Tibbs-Cortes, Dylan L. Schultz, Stephan Schmitz-Esser

**Affiliations:** 1 Department of Animal Science, Iowa State University, Ames, Iowa, USA; 2 Interdepartmental Microbiology Graduate Program, Iowa State University, Ames, Iowa, USA; The University of Arizona, Tucson, Arizona, USA

**Keywords:** *Listeria monocytogenes*, ST121, plasmid

## Abstract

We performed Oxford Nanopore and Illumina sequencing to generate accurate, closed genomes for the *Listeria monocytogenes* strains 6179 and L58-55. The new assemblies were generally similar to the previous Illumina-based assemblies, but additional rRNA operons and repeat regions were identified in the new assembly for strain 6179.

## ANNOUNCEMENT

*Listeria monocytogenes* is a gram-positive foodborne pathogen capable of causing severe infections and surviving antimicrobial conditions used to mitigate bacterial contamination in food production (1). Many *L. monocytogenes* strains belonging to sequence type (ST) 121 exhibit persistence, whereby a strain can be repeatedly isolated from the same food production environment over many years (2–5). The *L. monocytogenes* ST121 strain 6179 was originally isolated from a cheese production facility in Ireland, and its response to stressors encountered in food production has been extensively studied (6–11). Another ST121 strain, L58-55, was isolated from a human patient (12). Draft genomes for strains 6179 and L58-55 were previously generated using short-read sequencing (13, 14). Here, we utilized long- and short-read sequencing to obtain closed genomes for each strain.

6179 and L58-55 were stored in tryptic soy broth (TSB) with 20% glycerol at −80°C. Strains were grown in TSB at 37°C to OD_600_ ~2.5, and DNA was extracted using a Genomic-tip 20/G Kit (Qiagen). DNA samples were processed for library preparation without shearing and size selection using the SQK-LSK109 kit and the EXP-NBD104 barcoding kit; sequencing was conducted on an X5 flow cell using an Oxford Nanopore GridION with Guppy 4.3.4 for base-calling. Additionally, 6179 was sequenced on the Illumina MiSeq platform (250 bp, paired-end reads) after library preparation with the NEBNext Ultra II FS Kit. Available Illumina sequencing data from a previous MiSeq run (Illumina Nextera XT, 300 bp, paired-end reads; SRX16175649) was used for the L58-55 assembly (14).

For Illumina data, adapters were trimmed and low-quality reads were removed using BBDuk v37.36 with k, mink, and hdist values of 23, 11, and 1, respectively, with the tbo and tpe flags enabled. Quality trimming of both ends was performed with a minimum value of 20 (15). Hybrid assembly was then performed with processed Illumina data and raw long-read data in SPAdes 3.14.1 with the isolate option enabled (16). Contigs with k-mer coverage of less than 1 were discarded. Coverage statistics were calculated against the remaining contigs using the BBMap covstats option (15), and the circularity of the contigs was confirmed with Circlator v1.5.5 using default parameters (17). Genomes were annotated with PATRIC and the NCBI Prokaryotic Genome Annotation Pipeline (18–20). Percent nucleotide identities between the newly assembled contigs and the previous genomes were calculated using the NUCmer function of MUMmer 3.23 (21).

For both strains, sequencing and assembly yielded closed genomes consisting of two contigs, one representing the chromosome and one representing the plasmid. The average coverages, lengths, GC content, and sequencing statistics are shown in Table 1. The new assemblies were overall highly similar to the previous assemblies in terms of percent nucleotide identity (Table 1). However, the hybrid 6179 chromosome assembly is 26,072 bp longer than the previous version. This discrepancy was primarily due to two previously undetected rRNA operons and to multiple repeat regions primarily located within prophages (Table 1). This work adds two high-quality assemblies to the list of *L. monocytogenes* genomes.

**TABLE 1:** Statistics for the chromosome and plasmid assemblies of *L. monocytogenes* strains 6179 and L58-55

	6179 chromosome	6179 plasmid	L58-55 chromosome	L58-55 plasmid
Number of raw Illumina reads	6,449,280		1,266,616	
Average Illumina read length (bp)	250.0		298.5	
Number of raw Nanopore reads	62,132		56,185	
Average Nanopore read length (bp)	9,543		9,635	
Nanopore NC50 (bp)	25,250		25,250	
Average coverage	208.1	236.2	62.6	63.1
GC content (%)	37.95	36.54	37.98	36.54
Assembly length (bp)	3,036,692	61,196	2,975,970	61,167
Prior assembly length (bp)^a^	3,010,620	62,206	2,977,348	63,428
Percent nucleotide identity to prior assembly (%)	99.98	100	99.96	100
GenBank accession number	CP098509.1	CP098510.1	CP098507.1	CP098508.1
Illumina raw read accession number	SRX16203303		SRX16175649	
Nanopore raw read accession number	SRX16203304		SRX16175650	

^a^ The previous assemblies were based on Illumina sequencing only, and the genomes were not closed.

## Data Availability

The complete *L. monocytogenes* genome sequences have been deposited in GenBank with the BioProject accession numbers PRJEB1355 (6179) and PRJNA335730 (L58-55). GenBank and SRA accession numbers for each strain are listed in Table 1.
